# Regional heterogeneity and unexpectedly high abundance of *Cooperia punctata* in beef cattle at a northern latitude revealed by ITS-2 rDNA nemabiome metabarcoding

**DOI:** 10.1186/s13071-021-05137-y

**Published:** 2022-01-06

**Authors:** Eranga Lakshitha De Seram, Elizabeth Mary Redman, Felicity Kaye Wills, Camila de Queiroz, John Ross Campbell, Cheryl Lynne Waldner, Sarah Elizabeth Parker, Russell William Avramenko, John Stuart Gilleard, Fabienne Dominique Uehlinger

**Affiliations:** 1 University of Saskatchewan, Saskatoon, SK Canada; 2 University of Calgary, Calgary, AB Canada

**Keywords:** Beef herds, Cattle gastrointestinal nematodes, *Cooperia*, *C. punctata*, ITS-2 rDNA, Nemabiome metabarcoding, Northern latitudes

## Abstract

**Background:**

The species composition of cattle gastrointestinal nematode (GIN) communities can vary greatly between regions. Despite this, there is remarkably little large-scale surveillance data for cattle GIN species which is due, at least in part, to a lack of scalable diagnostic tools. This lack of regional GIN species-level data represents a major knowledge gap for evidence-based parasite management and assessing the status and impact of factors such as climate change and anthelmintic drug resistance.

**Methods:**

This paper presents a large-scale survey of GIN in beef herds across western Canada using ITS-2 rDNA nemabiome metabarcoding. Individual fecal samples were collected from 6 to 20 randomly selected heifers (*n* = 1665) from each of 85 herds between September 2016 and February 2017 and 10–25 first season calves (*n* = 824) from each of 42 herds between November 2016 and February 2017.

**Results:**

Gastrointestinal nematode communities in heifers and calves were similar in Alberta and Saskatchewan, with *Ostertagia ostertagi* and *Cooperia oncophora* being the predominant GIN species in all herds consistent with previous studies. However, in Manitoba, *Cooperia punctata* was the predominant species overall and the most abundant GIN species in calves from 4/8 beef herds*.*

**Conclusions:**

This study revealed a marked regional heterogeneity of GIN species in grazing beef herds in western Canada. The predominance of *C. punctata* in Manitoba is unexpected, as although this parasite is often the predominant cattle GIN species in more southerly latitudes, it is generally only a minor component of cattle GIN communities in northern temperate regions. We hypothesize that the unexpected predominance of *C. punctata* at such a northerly latitude represents a range expansion, likely associated with changes in climate, anthelmintic use, management, and/or animal movement. Whatever the cause, these results are of practical concern since *C. punctata* is more pathogenic than *C. oncophora*, the *Cooperia* species that typically predominates in cooler temperate regions. Finally, this study illustrates the value of ITS-2 rDNA nemabiome metabarcoding as a surveillance tool for ruminant GIN parasites.

**Graphical Abstract:**

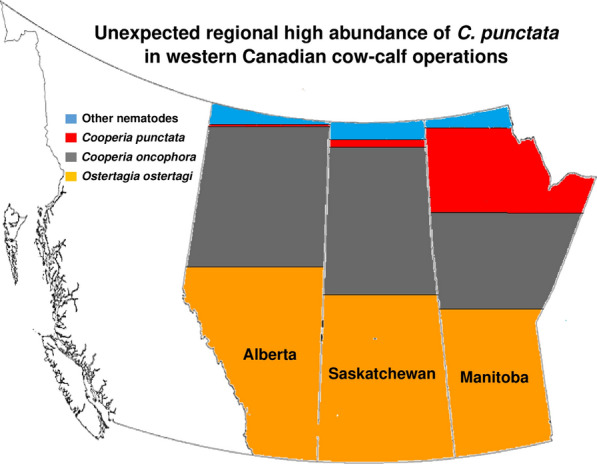

**Supplementary Information:**

The online version contains supplementary material available at 10.1186/s13071-021-05137-y.

## Background

Co-infection with multiple gastrointestinal nematode (GIN) species is common in cattle [1]. The infection intensities of the different GIN species will significantly influence the production impacts and the risk of clinical disease [2, 3]. The relative abundance of different GIN species is known to vary greatly at the macro-geographical level, with the most abundant species in northern temperature regions being distinctly different from those at more southerly latitudes [4]. Many factors can affect GIN species distribution and abundance, including climate, grazing management, and anthelmintic use [5–9]. However, there is minimal regional GIN species-level data in cattle from most parts of the world. This important knowledge gap is most likely due to the laborious and specialized nature of identifying GIN species in fecal samples using coproculture and microscopy [10]. The recent development of ITS-2 rDNA nemabiome metabarcoding provides a more accurate, objective, and scalable approach to examining GIN species composition of GIN parasite communities in grazing livestock [4, 11–13].

Previous work has shown that the two most abundant GIN species in cattle in northern temperate regions in North America, including western Canada, are *Ostertagia ostertagi* and *Cooperia oncophora,* with a number of other species being present at variable but typically low levels [4, 14]. *Ostertagia ostertagi* is the most pathogenic of the cattle GIN with the potential to cause severe clinical disease, but subclinical infections are more common at northern latitudes resulting in production loss such as reduced average daily gain and carcass quality [2, 3, 15]. *Cooperia oncophora* is less pathogenic but has become more concerned in recent years due to the high prevalence of macrocyclic lactone resistance [9, 16]. *Cooperia punctata*, the most pathogenic of the bovine *Cooperia* species, is typically found at high levels in more southerly regions but was recently reported in several beef cattle herds in eastern Canada, albeit as a minority of the GIN species overall [4].

In this paper, we report the application of ITS-2 nemabiome metabarcoding to survey the GIN communities in 85 beef herds across western Canada. We found an unexpectedly high relative abundance of *C. punctata* in Manitoba, revealing a distinct regional heterogeneity in GIN communities of cattle reared under relatively similar management and climatic conditions. The occurrence of *C. punctata* beyond what is generally considered its normal geographical range is of concern given its relatively high pathogenicity and propensity to develop macrocyclic lactone resistance.

## Methods

### Sample population

The cow-calf operations participating in this study were enrolled in the Western Canadian Cow-Calf Surveillance Network (WCCCSN). The formation of this network has previously been described [17, 18]. Briefly, producers for the WCCCSN were enrolled based on national agricultural census data to obtain a geographically representative sample population from western Canada, including Alberta, Saskatchewan, and Manitoba. Private veterinarians providing services to cow-calf operations in these provinces were asked to assist in recruiting producers. Inclusion criteria for participation were a minimum herd size of 100 cows, willingness to complete questionnaires regarding grazing systems, pasture management, and internal parasite control, and allowing the collection of biological samples from cattle. In situations where more producers were identified than needed from a particular region, the required number of producers was selected on a random basis. At the time of data collection for this study, there were 111 cow-calf operations enrolled in the WCCCSN (55 in Alberta, 35 in Saskatchewan, and 21 in Manitoba). Through a survey on parasite management distributed in the summer of 2016, producers were asked to collect fecal samples from their heifers during the fall pregnancy diagnosis. They were also asked to voluntarily collect fecal samples from their 2016 calf crop around weaning (fall 2016 and winter 2017) and submit them to the laboratory for processing [19].

### Fecal sample collection

Fecal sampling from heifers took place between September 2016 and February 2017. Each herd was provided with a sampling kit. The herd veterinarian was asked to collect fecal samples from the rectum of 20 randomly selected heifers based on the availability; if 20 heifers were not available, the youngest bred cows were sampled for a total of 20 samples per herd. This sampling strategy resulted in a median age of sampled heifers of 20 months (range 12–36 months). Calves were sampled from November 2016 to February 2017. Producers were instructed to collect at least two handfuls of feces (freshly voided or rectally collected) from 20 conveniently selected individual calves. The median age of calves sampled was eight months (range 7–9 months). The number of samples collected from each herd ranged from 6 to 20 samples (median 20) for heifers and 10 to 25 (median 20) for calves. Individual fecal samples from heifers and calves were collected into labeled plastic bags, with the air expelled, stored in an insulated container at room temperature, and shipped within 24 h of collection to the laboratory at the University of Saskatchewan, Saskatoon, Saskatchewan, Canada (heifer samples), or the laboratory at the University of Calgary, Calgary, Alberta, Canada (calf samples). Samples were received by respective laboratories within 24 to 96 h (median 48 h) of collection and stored at room temperature in the sealed bags in the insulated containers until fecal egg counting was performed.

There were 1655 heifer samples (Alberta = 876, Saskatchewan = 468, Manitoba = 311) from 85 herds (Alberta = 45, Saskatchewan = 24, Manitoba = 16) available to obtain fecal egg counts (FEC) and subsequent analysis. For the calves, 824 (Alberta = 447, Saskatchewan = 211, Manitoba = 166) fecal samples from 42 herds (Alberta = 23, Saskatchewan = 10, Manitoba = 9).

### Fecal egg counting

Individual fecal samples were processed within three to five days of collection. A modified Wisconsin sugar flotation technique with minor modifications was used to process fecal samples [20]. In brief, 5 and 3 g of feces were used to obtain FEC from heifers and calves, respectively. The fecal sample was mixed with 15 ml of tap water to create a homogenous slurry and filtered through a single layer of cheesecloth (grade 60) into a plastic cup. The filtrate was transferred into a 16 × 125 mm test tube and centrifuged (535×*g*, 10 min). The supernatant was carefully decanted, and the sediment was resuspended in Sheather's solution (specific gravity 1.27), leaving an approximately 5 mm air space from the top of the tube. The sample was again centrifuged (535×*g*, 10 min). Sheather’s solution was poured to a slight convex meniscus on the test tube, a coverslip was placed on top, and the sample was left to stand for 30 min. The coverslip was then carefully removed, placed on a glass slide, and observed under the microscope at ×100 total magnification. Gastrointestinal nematode eggs were identified as strongyle-type spp., *Nematodirus* spp., or *Trichuris* spp. The theoretical detection sensitivity of the test was 0.20 eggs per gram of feces (EPG) for heifers and 0.33 EPG for calves.

### Coproculture larval harvesting

Coprocultures were prepared at the same time as fecal egg counting. A different pooling strategy was used for the heifer and calf samples because the low number of larvae harvested per herd for the former did not allow meaningful quantitative data to be generated at the individual herd level.

#### Heifer samples

A modified coproculture protocol was used to harvest third-stage nematode larvae (L3) [21]. Briefly, a composite fecal sample was prepared for each herd by pooling 12 g of homogenized feces from each heifer in the herd, and three coprocultures were set up comprising 80 g of the composite feces mixed with vermiculite and tap water in a 250 ml glass. Cultures were incubated at room temperature (approximately 20–23 °C) for 21 days, after which time L3 were harvested, washed twice in tap water by centrifugation at 3725×*g* for 3 min before resuspending in 0.3 ml of tap water, and fixed by the addition of 0.7 ml of 95% ethanol. The larvae derived from the three coprocultures of composite fecal samples per herd were then pooled to provide one pool of harvested larvae per herd. After the enumeration of an aliquot of larvae by microscopy, samples were sent to the University of Calgary laboratory, where they were kept frozen at −80 °C for archiving and ITS-2 rDNA nemabiome metabarcoding. Since there were insufficient larvae obtained for meaningful quantitative analysis at the individual herd level, the L3 harvested from each herd were put into two pools per province; one pool for small herds (≤ 300 cow-calf pairs) and one pool for large herds (> 300 cow-calf pairs). The small herd pools comprised 29, 16, and 10 herds, and the large herd pools comprised 15, 8, and 5 herds for Alberta, Saskatchewan, and Manitoba, respectively (larvae were not available for pooling from one large herd from Alberta and one small herd from Manitoba). This pooling strategy was chosen to give some degree of replication within the limitations of the small number of larvae harvested from many individual herds and compare the species abundance between herds of different sizes and between provinces. The total number of L3 in the small and large herd pools, respectively, were as follows: Alberta = 4200 and 3900 L3, Saskatchewan = 3600 and 4400 L3, Manitoba = 3800 and 5200 L3. Three separate aliquots of 1000 L3 were then taken from each pool to prepare triplicate genomic DNA samples for nemabiome metabarcoding.

#### Calf samples

For calves, individual animal coprocultures were prepared from 40 g of homogenized feces as per the method of Roberts and O’Sullivan [21]. Culture conditions and harvesting procedures were similar to those described for the heifer samples. Larvae were counted by microscopy, and 50% of the larvae from individual samples were pooled to create a single herd-level pool of larvae. Pooled L3 were washed, centrifuged, enumerated, and fixed in 95% ethanol, similar to the procedure described for heifers. Samples were stored at −80 °C until being processed for ITS-2 rDNA nemabiome metabarcoding.

There were adequate L3 counts for quantifying GIN species proportions at the herd level for 40 out of 42 herds sampled (*n* = 22, 10, and 8) in Alberta, Saskatchewan, and Manitoba, respectively). Genomic DNA prepared from 250 L3 from each herd-level pool was used for ITS-2 rDNA sequencing. There were sufficient larvae in most herd-level pools for two or three separate aliquots of 250 L3 to prepare duplicate or triplicate genomic DNA samples. However, four herd-level pools had insufficient larvae for duplicate samples; consequently, a single aliquot of 250 L3 was processed (Additional file 1: Figure S1).

### ITS-2 rDNA nemabiome metabarcoding

The ITS-2 rDNA nemabiome metabarcoding methodology has been previously described and validated [11]. Protocol details are available at https://www.nemabiome.ca/sequencing.html [22]. Briefly, larvae were placed in a Proteinase K (120 μg/ml) lysis buffer (50 mM KCl, 10 mM Tris (pH 8.3), 2.5 mM MgCl_2_, 0.45% Nonidet P-40, 0.45% Tween 20, 0.01% (w/v) gelatin) to create pooled crude lysates. Molecular-grade double-distilled water was used to make 1:10 dilutions of the pooled crude lysates used as a template for first-round PCR amplification of the ITS-2 rDNA target (311–331 bp fragment) as described in Avramenko et al. [11]. Following purification with AMPure XP Magnetic Beads (1×) (Beckman Coulter Inc., Indianapolis, IN, USA), Illumina indices and P5/P7 sequencing tags (Illumina, Inc., San Diego, CA, USA) were added using limited cycle PCR amplification, and the final amplicon products purified using the same method as above. Approximately 50 ng of amplicon were pooled from each sample to make up the master sequencing library, quantified using the KAPA qPCR Library Quantification Kit (Roche/Kapa Biosystems, Inc., Wilmington, MA, USA). The final concentration of the pooled library was 12.5 nM, with the addition of 25% PhiX Control v3 (Illumina, FC-110-3001), and it was run on an Illumina MiSeq Desktop Sequencer using a 500-cycle paired-end reagent kit (MiSeq Reagent Kits v2, MS-103-2003). Utilizing the Mothur software package, a bioinformatics pipeline was used to assign nematode species identity to each sequenced read using previously described methods [4]. Further details of the pipeline are available at https://www.nemabiome.ca/analysis.html [23]. Sequence reads were multiplied by previously validated correction factors specific to individual GIN species [11]. The number of sequence reads mapping to each species reference sequence was divided by the total number of mapped reads per sample to determine the percentage species composition of each sample. The total sequence read number mapping to ITS-2 rDNA reference sequences for each sample ranged from 13,401 to 32,801 reads for heifer samples and 15,178 to 83,935 reads for calf samples.

### Data analyses

At the individual animal level, the proportions of individual fecal samples [95% confidence interval (CI)] positive for strongyle-type, *Nematodirus* spp., and *Trichuris* spp. were determined for calves and heifers based on identifying at least one GIN egg under the microscope. The overall arithmetic mean EPG [± standard deviation (SD)] of the three morphologically different GIN egg types were also calculated for both heifers and calves. For calves, the herd-level arithmetic mean EPG (±SD) of all GIN egg types was calculated and presented with the relative herd-level GIN species proportions.

At the provincial level, the arithmetic mean EPG (±SD) of strongyle-type FEC for each province was calculated, and the statistical difference between provinces was determined using a generalized estimating equation model with a negative binomial distribution and log link function, accounting for clustering at the herd-level for both calves and heifers. *Nematodirus* spp. and *Trichuris* spp. FEC were very low in both calves and heifers; therefore, their provincial-level arithmetic means were not estimated.

Alpha diversity was calculated to determine the overall species diversity of GIN populations in calves and heifers within a province. For calves, species diversity data of analytical replicates (i.e., 250 L3 aliquots; 62, 36, and 20 aliquots for Alberta, Saskatchewan, and Manitoba, respectively) of herd-level pools were used for the mean comparison. For heifers, species diversity data of analytical replicates (i.e., 1000 L3 aliquots; 12 aliquots per province) of each provincial pool were used for the mean comparison. Analytical replicates for heifers were meaningful because they represent multiple herds within a province as larvae from many individual herds went into each provincial pool. The calculations were performed in Mothur v.1.36.1 using the built-in inverse Simpson calculation as previously reported [11]. To assess whether the inverse Simpson index differed significantly between each province, a one-way ANOVA, assuming non-equal variances, was performed using a Games–Howell post hoc comparison in SPSS statistical software (IBM Corp. Released 2012. IBM SPSS Statistics for Macintosh, Version 21.0. Armonk, NY, USA).

Beta diversity estimation was performed for three major species, *O. ostertagi*, *C. oncophora*, and *C. punctata*, using the MetaStats plugin in 275 Mothur v. 1.36.1, using 1000 permutations and default parameters to determine whether the species composition differed in calves and heifers between two different provinces [24]. MetaStats assumed that the data were not normally distributed; therefore, modified non-parametric t-tests (two-tailed) for pairwise comparisons of beta diversity estimations of each GIN species in each province were used [24]. This method can overestimate the GIN species with a lower abundance; therefore, significance was not claimed if the species were present at less than 2% on average in both comparable groups. Significance was declared if *P* < 0.05. In terms of calves, the arithmetic mean FEC of strongyle-type spp., *Nematodirus* spp., and *Trichuris* spp. for each herd were reported with the relative GIN species proportions in each sample.

## Results

### Fecal egg count and ITS-2 rDNA nemabiome metabarcoding data for gastrointestinal nematodes in heifers

Strongyle-type eggs were detected in 92.0% (95% CI 91.0–93.0) of individual heifer samples and 100% of herds with an overall mean FEC of 5.3 (SD = 7.7) EPG (range = 0–92). *Nematodirus* spp. eggs were detected in 1.8% (95% CI 1.0–3.0) of individual samples and 24.7% (21/85) of herds with an overall mean FEC of 0.01 (SD = 0.1) EPG (range = 0–2.4). *Trichuris* spp. eggs were detected in 1.0% of individual samples (95% CI 1.0–2.0) and 21.2% (18/85) of herds with an overall mean FEC of 0.003 (SD = 0.3) EPG (range = 0–0.6). The mean strongyle-type FEC of heifers in Alberta, Saskatchewan, and Manitoba were 5.1 (SD = 6.5, range = 0–55.6), 5.9 (SD = 8.7, range = 0–92), and 5.2 (SD = 9.3, range = 0–85), respectively, and they were not significantly different (Wald *χ*^2^ = 0.66, number of observations = 1655, number of groups = 85, *P* = 0.719).

**Fig. 1:**
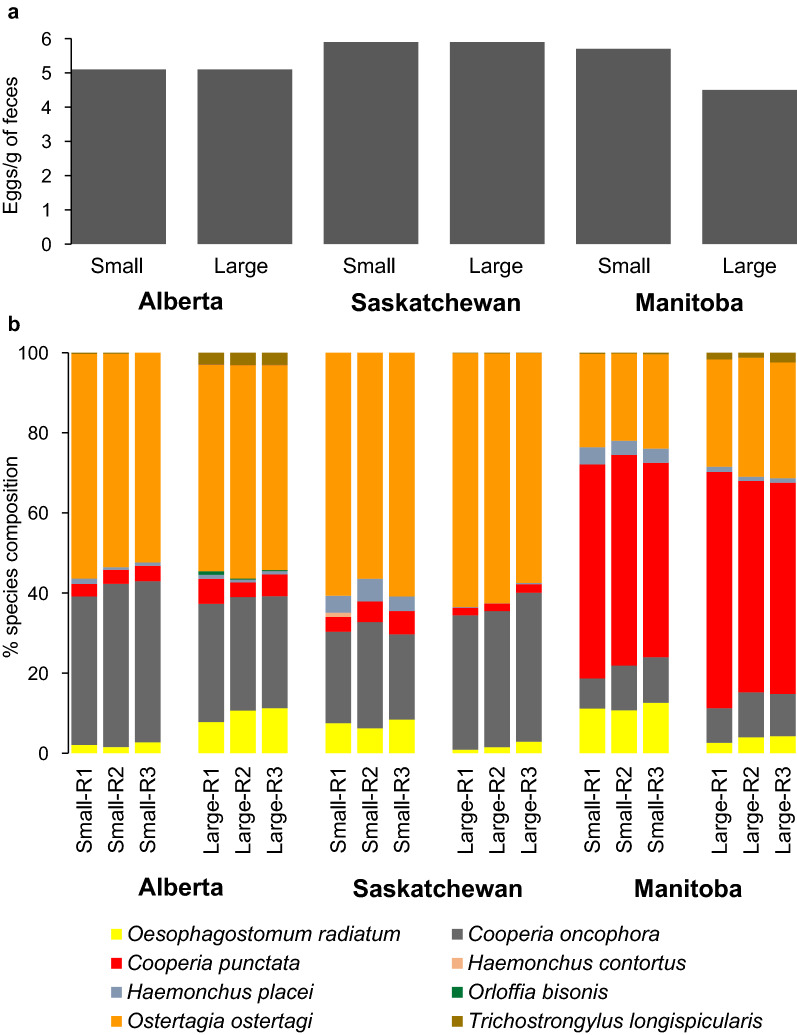
Fecal egg counts and gastrointestinal nematode species proportions of heifer fecal sample pools from western Canadian beef farms. Samples were pooled at the provincial level with two pools per province categorized based on herd size (small ≤ 300 cow-calf pairs, large > 300 cow-calf pairs). Three independent aliquots (R1, R2, R3) of 1000 third-stage larvae were taken for ITS-2 rDNA nemabiome metabarcoding for each pool. **a** represents the arithmetic mean of individual animal strongyle-type fecal egg counts of each provincial pool. **b** indicates the relative gastrointestinal nematode species proportions determined by ITS-2 rDNA nemabiome metabarcoding of third-stage larvae pooled by herd size and province

ITS-2 rDNA nemabiome metabarcoding revealed that *O. ostertagi* was the predominant parasite species in heifers from Alberta and Saskatchewan herds (Fig. 1), with an overall relative abundance of 53.9 and 52.0% in Alberta small and large herds, respectively, and 59.3 and 61.0% in Saskatchewan small and large herds, respectively. *Cooperia oncophora* was the second most abundant GIN in those two provinces, making up 39.4 and 28.6% of the parasite species proportions overall from small and large herds in Alberta, respectively, and 23.5 and 34.9% of the GIN populations from small and large herds in Saskatchewan, respectively. In contrast, *Cooperia punctata* was the predominant GIN species in Manitoba, with an overall relative abundance of 51.5 and 54.8% in small and large herds, respectively. *Ostertagia ostertagi* was the second most abundant GIN in Manitoba's small and large herds, with an overall relative abundance of 22.9 and 28.4%, followed by *C. oncophora* with an overall relative abundance of 10.0 and 10.2% in those herds, respectively. Other GIN species were present in smaller proportions in Alberta, Saskatchewan, and Manitoba regional pools, including *Oesophagostomum radiatum* (4.1–7.4%), *Haemonchus placei* (0.9–2.4%), *Trichostrongylus longispicularis* (0.1–1.6%), *Haemonchus contortus* (0–0.2%), and *Orloffia bisonis* (0–0.3%).

The comparison of inverse Simpson index for alpha diversity manifested a significant difference in overall GIN species diversity between provinces (*F*_(2, 33)_ = 27.3, *P* < 0.001). The post hoc comparisons of the inverse Simpson indexes revealed that the overall GIN species diversity in heifers was greater in Manitoba (mean index = 3.60, SD = 0.64) herds than the Alberta (mean index = 2.54, SD = 0.32) herds (*P* = 0.037). Similarly, the overall GIN species diversity of Manitoba herds was greater than Saskatchewan herds (mean index = 2.31, SD = 0.36) herds (*P* = 0.009). Nevertheless, Alberta heifers’ alpha GIN species diversity was not significantly different from Saskatchewan heifers (*P* = 0.765). Beta species diversity comparisons revealed that the relative abundance of *C. punctata* was significantly greater in heifers from Manitoba than Alberta (*t* = 35.1, *P* < 0.001) and Saskatchewan (*t* = 33.6, *P* < 0.001) (Table 1). Gastrointestinal nematode populations in Alberta had a significantly greater abundance of *C. oncophora* (*t* = 12.1, *P* < 0.001) and *O. ostertagi* (*t* = 27.5, *P* < 0.001) than in Manitoba. Similarly, *C. oncophora* (*t* = 10.0, *P* < 0.001) and *O. ostertagi* (*t* = 26.7, *P* < 0.001) abundances were significantly greater in Saskatchewan than Manitoba populations. Significant differences were not assessed for *Nematodirus helvetianus*, *O. bisonis*, *H. placei*, *O. radiatum*, and *Trichostrongylus axei* due to their low relative abundance (< 2%).

**Table 1:** Beta-diversity (MetaStats) significance for individual parasite species of gastrointestinal nematodes in provincial level larval pools from heifers between 12 and 36 months of age from 83 cow-calf operations in Alberta (AB; *n* = 44), Saskatchewan (SK; *n* = 24) and Manitoba (MB; *n* = 15), Canada

Nematode species	Mean % (±SD)^a^			Statistical analysis		
	AB	SK	MB	AB vs SK	AB vs MB	SK vs MB
*Ostertagia ostertagi*	53.6 (1.8)	60.0 (3.3)	24.8 (3.2)	*t* = 5.864 *P* < 0.001*	*t* = 27.480 *P* < 0.001*	*t* = 26.708 *P* < 0.001*
*Cooperia oncophora*	32.7 (6.1)	29.3 (6.3)	9.6 (2.5)	*t* = 1.349 *P* = 0.190	*t* = 12.110 *P* < 0.001*	*t* = 10.006 *P* < 0.001*
*Cooperia punctata*	4.0 (1.7)	3.5 (2.4)	54.0 (4.6)	*t* = 0.590 *P* = 0.561	*t* = 35.089 *P* < 0.001*	*t* = 33.620 *P* < 0.001*

^a^Standard deviation

*Statistically significant

### Fecal egg count and ITS-2 rDNA nemabiome metabarcoding data for gastrointestinal nematodes in calves

Strongyle-type eggs were detected in 91.1% (95% CI 89.0–93.0%) of individual calf samples and 100% of herds with an overall mean FEC of 17.9 (SD = 20.3) (range = 0–178). *Nematodirus* spp. eggs, were detected in 30.7% (95% CI 27.6–34.0) of individual calf samples and 100% of herds with an overall mean FEC of 0.8 (SD = 2.9) EPG (range = 0–36). *Trichuris* spp. eggs were detected in 33.1% (95% CI 29.9–36.5) of individual calf samples and 85.7% (36/42) of herds with an overall mean FEC of 0.6 (SD = 2.0) EPG (range = 0–32 EPG). Mean strongyle-type FEC in calves from Alberta, Saskatchewan, and Manitoba were 15.3 (SD = 15.8, range = 0–88), 17.0 (SD = 16.2, range = 0–86), and 24.7 (SD = 31.0, range = 0–178), respectively, with no significant differences between those egg counts (Wald *χ*^2^ = 2.27, number of observations = 844, number of groups = 42, *P* = 0.322).

**Fig. 2:**
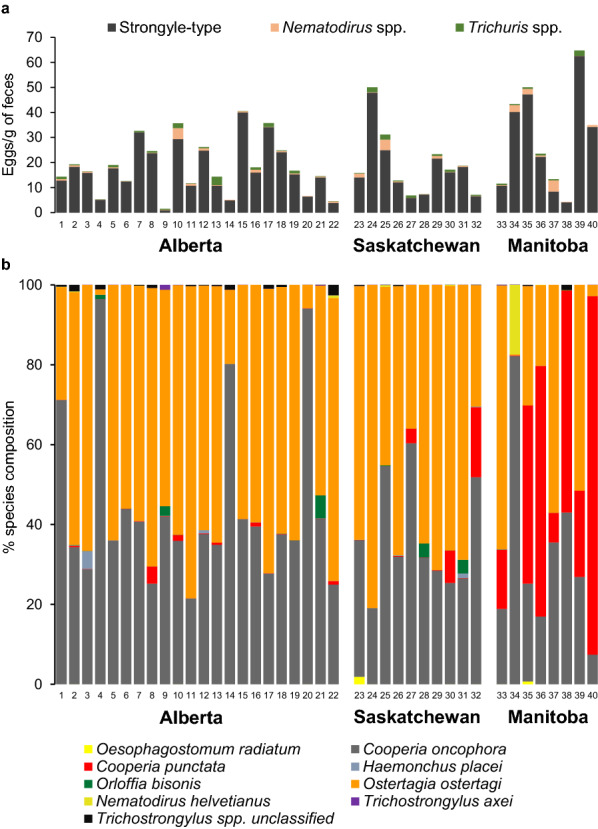
Fecal egg counts and gastrointestinal nematode species proportions in fecal samples of calves from 40 western Canadian cow-calf operations. Fecal samples from 10 to 20 individual calves were pooled at the herd level. Figure **a** represents the arithmetic means of 10–20 individual calf fecal egg counts of strongyle-type, *Nematodirus* spp. and *Trichuris* spp. eggs in each herd. Figure **b** represents the relative GIN species proportions of each herd determined by ITS-2 rDNA nemabiome metabarcoding of herd-level third-stage larval pools. Numbers from 1 to 40 on the *x*-axis identify the individual herds. Except for herds 9, 14, 25, and 34, nemabiome metabarcoding was undertaken on duplicate or triplicate aliquots of 250 larvae per herd (Additional file 1: Figure S1)

ITS-2 rDNA nemabiome metabarcoding revealed that *O. ostertagi* was the most abundant species in Alberta and Saskatchewan, with a mean overall relative abundance of 50.8 (SD = 22.4, range = 1.4–78.2) and 56.0% (SD = 16.9, range = 30.4–80.9), respectively. It was the predominant species in 18/22 herds in Alberta and 7/10 herds in Saskatchewan (Fig. 2). *Cooperia oncophora* was the second most abundant GIN species in those two provinces, with a mean overall abundance of 44.2 (SD = 21.3, range = 21.5–96.4) and 36.4% (SD = 14.0, range = 19.0–60.4) in Alberta and Saskatchewan, respectively. It was the predominant species in 4/22 Alberta herds and 2/10 Saskatchewan herds. In Alberta and Saskatchewan, *C. punctata* abundance was relatively low with a respective mean overall abundance of 0.2 (SD = 0.4, range = 0–4.3) and 2.3% (SD = 3.5, range = 0.02–17.5). However, in Manitoba, *C. punctata* was the most abundant GIN species, with a mean overall relative abundance of 28.2% (SD = 26.2, range = 0.1–89.8). *Cooperia punctata* was the predominant species in 4/8 of Manitoba herds. The mean relative species abundances of *O. ostertagi* and *C. oncophora* in Manitoba were 34.7% (SD = 28.4, range = 7.4–82.2) and 31.9 (SD = 23.1, range = 0.07–66.1), respectively. The former was the predominant species in 3/8, and the latter was the predominant species in 1/8 herds from Manitoba. *Nematodirus helvetianus* (0.07–2.2%), *O. bisonis* (0–0.7%), *H. placei* (0.02–0.3%), *O. radiatum* (0–0.2%), and *T. axei* (0–0.07%) were also present in some herds, but their relative abundances were very small.

The comparisons of the inverse Simpson index revealed a significant difference in overall GIN species diversity between provinces (*F*_(2, 116)_ = 8.59, *P* < 0.001). The post hoc comparisons of the inverse Simpson indexes indicated an overall lower GIN species diversity in the calves from Alberta (mean index = 1.78, SD = 0.34) herds than Manitoba (mean index = 2.15, SD = 0.55) herds (*P* = 0.025). In parallel, the overall GIN species diversity in Alberta’s calves was lower than in Saskatchewan (mean index = 1.93, SD = 0.20) calves (*P* = 0.024). However, there was no difference in the overall species alpha diversity between the Manitoba and Saskatchewan GIN populations (*P* = 0.219). Beta diversity analysis manifested that Manitoba farms had a significantly greater abundance of *C. punctata* than Alberta (*t* = 4.8, *P* = 0.001) and Saskatchewan (*t* = 4.4, *P* = 0.001) (Table 2). The relative abundance of *C. punctata* was significantly lower in Alberta herds than in Saskatchewan herds (*t* = −3.6, *P* = 0.001). Gastrointestinal nematode populations in Alberta herds had a significantly greater abundance of *C. oncophora* than GIN populations found on farms from Manitoba (*t* = 2.5, *P* = 0.022) and Saskatchewan (*t* = 2.1, *P* = 0.044). Furthermore, *O. ostertagi* was significantly lower in GIN populations found in Manitoba farms compared to GIN populations sampled in Alberta (*t* = −2.4, *P* = 0.023) and Saskatchewan farms (*t* = −3.2, *P* = 0.003). Statistical differences were not assessed for *N. helvetianus*, *O. bisonis*, *H. placei*, *O. radiatum*, and *T. axei* due to their low relative abundance (< 2%).

**Table 2:** Beta-diversity (MetaStats) significance for individual parasite species of gastrointestinal nematodes in herd level larval pools from calves < 1 year of age from 40 cow-calf operations in Alberta (AB; *n* = 22), Saskatchewan (SK; *n* = 10) and Manitoba (MB; *n* = 8), Canada

Nematode species	Mean % (±SD)^a^			Statistical analysis		
	AB	SK	MB	AB vs SK	AB vs MB	SK vs MB
*Ostertagia ostertagi*	50.8 (22.4)	56.0 (16.9)	34.7 (27.2)	*t* = 1.294 *P* = 0.188	*t* = 2.396 *P* = 0.024*	*t* = 3.173 *P* = 0.003*
*Cooperia oncophora*	44.2 (21.3)	36.4 (14.0)	31.9 (23.1)	*t* = 2.046 *P* = 0.044*	*t* = 2.496 *P* = 0.022*	*t* = 0.959 *P* = 0.352
*Cooperia punctata*	0.2 (0.94)	2.3 (3.5)	28.2 (26.2)	*t* = 3.614 *P* = 0.001*	*t* = 4.788 *P* = 0.001*	*t* = 4.409 *P* = 0.001*

^a^Standard deviation

*Statistically significant

## Discussion

There is a lack of large-scale studies exploring regional differences of GIN species distribution and abundance in cattle, with most existing information being largely historical or anecdotal. This paucity of studies is partly because of the specialist expertise and time required for species identification and the lack of scalable tools for quantifying parasite species abundance from fecal samples. The recent development of ITS-2 rDNA nemabiome sequencing now provides a powerful new approach to investigate the relative abundance of GIN species [11]. A recent ITS-2 rDNA metabarcoding study of 50 beef herds across Canada determined that *O. ostertagi* and *C. oncophora* were the two most predominant GIN species overall, consistent with the general understanding that these two species are well adapted to cold temperate climates [4, 14]. The only aspect of that study that suggested regional differences was the finding that *C. punctata* was present at a somewhat greater abundance overall in eastern Canada than in the West [4]. However, that species was still much less abundant than *O. ostertagi* and *C. oncophora* in the eastern Canadian herds overall. Here we have extended that work to explore potential regional differences in GIN species distribution and abundance in a larger scale study of cow-calf herds across three western Canadian provinces; Alberta (45 herds), Saskatchewan (24 herds), and Manitoba (16 herds).

One of the challenges of working with cattle GIN in northern latitudes is the typically low fecal egg excretion and the consequent low recovery of eggs or larvae from fecal samples, particularly in adult cattle. Consequently, a pooling strategy was taken to provide a regional overview of the GIN species abundance in western Canadian beef operations in which samples from heifers were pooled at the provincial level (two pools per province based on herd size categorization), and samples from calves were pooled at the herd level. The ITS-2 rDNA nemabiome metabarcoding results were consistent with the previous study of Canadian beef calves finding that *O. ostertagi* was the predominant GIN species in both Alberta and Saskatchewan cow-calf operations, with the next most abundant species being *C. oncophora* [4]. However, for Manitoba, the results were markedly different from Alberta and Saskatchewan, with a very high abundance of *C. punctata* across multiple herds. This species was the most abundant GIN species in both the heifer and calf samples from Manitoba overall and was the single most abundant GIN species in calves from 4/8 herds sampled from Manitoba. This finding was surprising because *C. punctata* is considered poorly adapted to cooler climates and has only been reported to occur at high abundance at more southerly latitudes such as mid-west, southern USA, and southern America, where it commonly dominates GIN communities [4, 9].

**Fig. 3:**
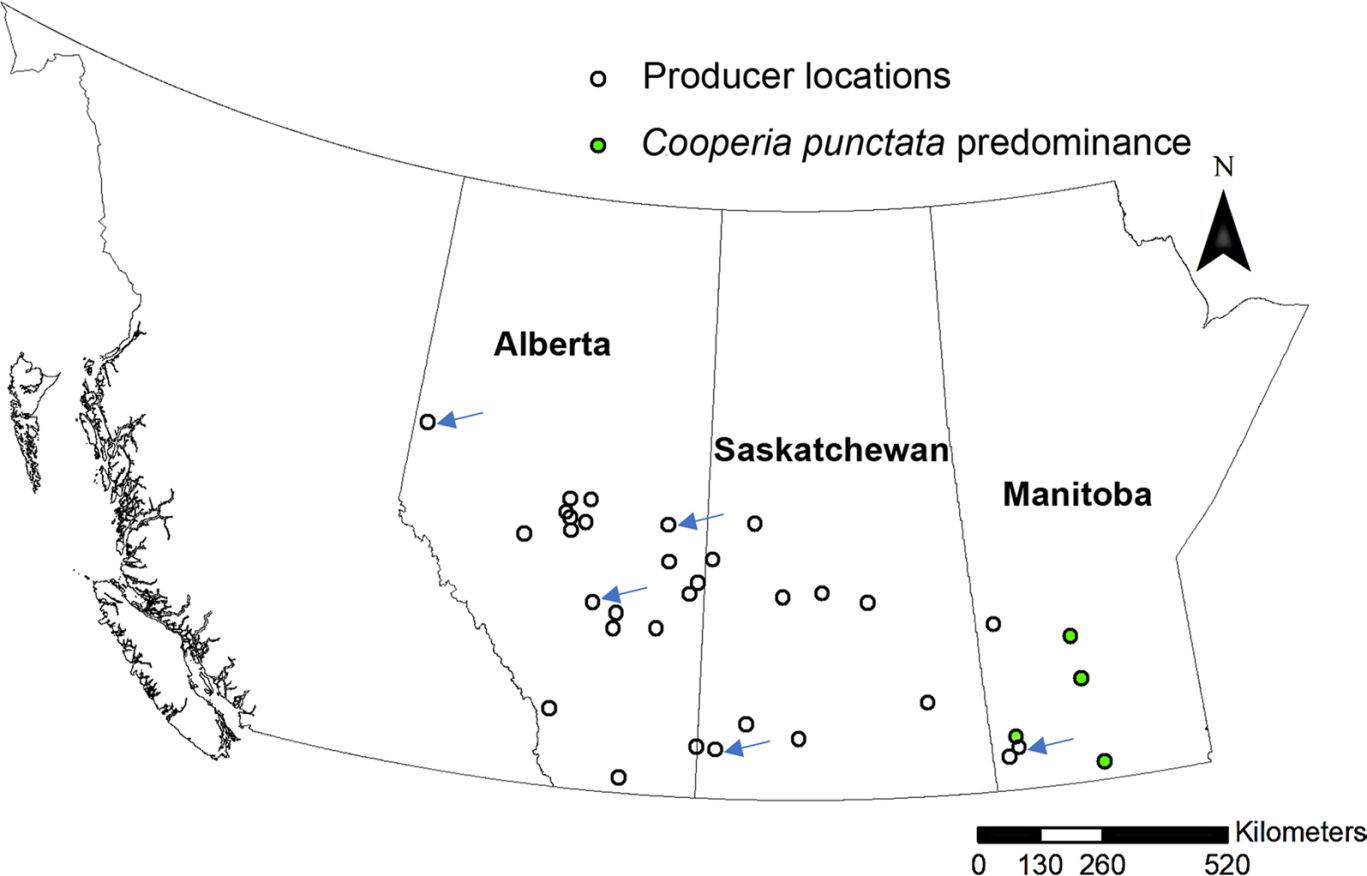
Approximate locations of 40 cow-calf operations where ITS-2 rDNA nemabiome metabarcoding data in fecal samples were available for calves. Those herds with a predominance of *Cooperia punctata* are indicated in green. Each location identified by an arrow contains two farms with the same postal code

The high abundance of *C. punctata* at such a northerly latitude is concerning since this species is significantly more pathogenic than *C. oncophora*, the species of *Cooperia* that generally predominates in cattle in cooler temperate regions such as western Canada. Research in experimentally infected beef calves shows that *C. punctata* negatively impacts appetite and nutrient utilization resulting in significant reductions in average daily gain and dry matter intake [25]. The lack of historical data and detailed regional studies of GIN abundance in North American beef herds over the last few decades means it is not certain whether the high abundance of *C. punctata* in Manitoba represents a change and, if so, how recently this might have occurred. Nevertheless, this parasite species has not been frequently reported at such northerly latitudes in the past, and to our knowledge, never as the predominant GIN species. Therefore, there is a strong possibility that these results reflect a range expansion of this species due to several factors. Trichostrongylid GIN are potentially sensitive to climate change since the part of the life cycle outside the host is very temperature- and moisture-dependent [26]. Range expansion associated with climate change has been suggested for several parasitic nematode species in the strongylid group. For example, the ruminant GIN nematode *H. contortus* is currently much more common in western Canadian and UK sheep farms than in previous decades, and modeling studies suggest that significant range expansion is likely to occur in northern Europe in the coming decades [27, 28]. A major concern is the lengthening of the grazing season predicted in Canada associated with global warming, which could lead to range expansion of GIN more usually found in southern latitudes [29]. Changes in cattle GIN parasite distribution could also be associated with the selection pressure for routinely used anthelmintics, particularly macrocyclic lactones. Ivermectin resistance is now widespread in *Cooperia* spp. in North America, and a study in the USA in 2015 suggested that the overall prevalence of *Cooperia* spp. was increasing due to the selection pressure for macrocyclic lactone resistance [9, 16]. Whatever the explanation of the unexpectedly high relative abundance of *C. punctata* in Manitoba cow-calf herds, its regional distribution in western Canada is not simply related to the latitude (Fig. 3). The localized distribution in Manitoba may suggest a single or small number of introduction events into that region, perhaps associated with the importation of cattle from particular sources further south. It will be interesting to map the distribution of *C. punctata* in Manitoba in more detail and investigate potential risk factors for its presence and the history of animal movements and importations in the region. It is also noteworthy that the sampling timing was not markedly different between provinces and so not the reason for the differences in the GIN community composition. However, all samples from heifers and calves were collected in the fall and winter. Therefore, a more detailed temporal study is warranted both across the grazing season and between years to understand further the relative abundance and the regional distribution of this parasite.

*Cooperia punctata* is also one GIN species that most commonly develop resistance to macrocyclic lactones, with several USA cattle reports [30, 31]. Ivermectin is currently the most widely used macrocyclic lactone in western Canadian beef operations [19]. We have recently confirmed ivermectin resistance in *C. oncophora*, *H. placei,* and *C. punctata* and provided evidence for ivermectin resistance of hypobiotic larvae of *O. ostertagi* in western Canada using integrated ITS-2 rDNA nemabiome metabarcoding and FEC reduction test results [32]. This finding suggests the emergence of ivermectin resistance in multiple GIN species in western Canada, including *C. punctata*.

## Conclusions

In summary, this study has revealed striking regional differences in GIN species abundance in western Canada. Specifically, a previously unidentified high relative abundance of *C. punctata* in beef cattle in Manitoba. The lack of previous reports of this parasite species at such a northerly latitude raises the possibility that its range is geographically expanding in North America, perhaps under the influence of climate change, anthelmintic use, animal movement, and/or other as yet undetermined management factors. The emergence of *C. punctata* as a major constituent of cattle parasite communities in northern latitudes is potentially problematic given the high pathogenicity and capacity for anthelmintic resistance in this GIN species. This study also provides an excellent illustration of the value of ITS-2 rDNA nemabiome metabarcoding as a surveillance tool for ruminant GIN parasites.

## Supplementary Information

**Additional file 1:** **Figure S1.** Gastrointestinal nematode species proportions determined by ITS-2 rDNA nemabiome metabarcoding of herd-level third-stage larval pools prepared from fecal samples of calves from 40 western Canadian cow-calf operations. Numbers from 1–40 on the *x*-axis identify the individual herds of each province. Except for herds 9, 14, 25, and 34, nemabiome metabarcoding was undertaken on duplicate or triplicate (R1, R2, R3) aliquots of 250 larvae per herd.

## Data Availability

The raw Fastq sequencing files generated during the current study are available in the NCBI database under the BioProject accession PRJNA788677 (https://www.ncbi.nlm.nih.gov/sra/PRJNA788677). Other datasets used are available from the corresponding authors on reasonable request.

## Abbreviations

CI: Confidence interval
EPG: Eggs per gram of feces
FEC: Fecal egg count(s)
GIN: Gastrointestinal nematode(s)
L3: Third-stage nematode larvae
SD: Standard deviation
WCCCSN: Western Canadian Cow-Calf Surveillance Network

## References

[1] Sutherland I, Scott I (2010). Gastrointestinal nematodes of sheep and cattle: biology and control.

[2] Myers GH, Taylor RF (1989). Ostertagiasis in cattle. J Vet Diagn Invest.

[3] Hawkins JA (1993). Economic benefits of parasite control in cattle. Vet Parasitol.

[4] Avramenko RW, Redman EM, Lewis R, Bichuette MA, Palmeira BM, Yazwinski TA (2017). The use of nemabiome metabarcoding to explore gastrointestinal nematode species diversity and anthelmintic treatment effectiveness in beef calves. Int J Parasitol.

[5] Bransby DI (1993). Effects of grazing management practices on parasite load and weight gain of beef cattle. Vet Parasitol.

[6] Stromberg BE, Averbeck GA (1999). The role of parasite epidemiology in the management of grazing cattle. Int J Parasitol.

[7] Fox NJ, Marion G, Davidson RS, White PCL, Hutchings MR (2015). Climate-driven tipping-points could lead to sudden, high-intensity parasite outbreaks. R Soc Open Sci.

[8] Gethings OJ, Rose H, Mitchell S, Dijk JV, Morgan ER (2015). Asynchrony in host and parasite phenology may decrease disease risk in livestock under climate warming: *Nematodirus battus* in lambs as a case study. Parasitology.

[9] Stromberg BE, Gasbarre LC, Ballweber LR, Dargatz DA, Rodriguez JM, Kopral CA (2015). Prevalence of internal parasites in beef cows in the United States: results of the national animal health monitoring system's (NAHMS) beef study, 2007–2008. Can J Vet Res.

[10] van Wyk JA, Cabaret J, Michael LM (2004). Morphological identification of nematode larvae of small ruminants and cattle simplified. Vet Parasitol.

[11] Avramenko RW, Redman EM, Lewis R, Yazwinski TA, Wasmuth JD, Gilleard JS (2015). Exploring the gastrointestinal “Nemabiome”: deep amplicon sequencing to quantify the species composition of parasitic nematode communities. PLoS ONE.

[12] Avramenko RW, Redman EM, Melville L, Bartley Y, Wit J, Queiroz C (2019). Deep amplicon sequencing as a powerful new tool to screen for sequence polymorphisms associated with anthelmintic resistance in parasitic nematode populations. Int J Parasitol.

[13] Redman E, Queiroz C, Bartley DJ, Levy M, Avramenko RW, Gilleard JS (2019). Validation of ITS-2 rDNA nemabiome sequencing for ovine gastrointestinal nematodes and its application to a large scale survey of UK sheep farms. Vet Parasitol.

[14] Hildreth MB, McKenzie JB (2020). Epidemiology and control of gastrointestinal nematodes of cattle in northern climates. Vet Clin North Am Food Anim Pract.

[15] Fox MT, Gerrelli D, Pitt SR, Jacobs DE, Gill M, Gale DL (1989). *Ostertagia ostertagi* infection in the calf: effects of a trickle challenge on appetite, digestibility, rate of passage of digesta and liveweight gain. Res Vet Sci.

[16] Kaplan RM (2020). Biology, epidemiology, diagnosis, and management of anthelmintic resistance in gastrointestinal nematodes of livestock. Vet Clin North Am Food Anim Pract.

[17] Moggy M, Pajor E, Thurston W, Parker S, Greter A, Schwartzkopf-Genswein K (2017). Attitudes of western Canadian cow-calf producers towards the code of practice for the care and handling of beef cattle. Can Vet J.

[18] Waldner CL, Parker S, Gesy KM, Waugh T, Lanigan E, Campbell JR (2017). Application of direct polymerase chain reaction assays for *Campylobacter fetus* subsp. *venerealis* and *Tritrichomonas foetus* to screen preputial samples from breeding bulls in cow-calf herds in western Canada. Can J Vet Res.

[19] Wills F, Campbell J, Parker S, Waldner C, Uehlinger F (2020). Gastrointestinal nematode management in western Canadian cow-calf herds. Can Vet J.

[20] Ito S (1980). Modified Wisconsin sugar centrifugal-flotation technique for nematode eggs in bovine feces. J Jpn Vet Med Assoc.

[21] Roberts FHS, O'Sullivan PJ (1950). Methods for egg counts and larval cultures for strongyles infesting the gastrointestinal tract of cattle. Aust J Agric Res.

[22] Nemabiome. 2021. https://www.nemabiome.ca/sequencing.html. Accessed 20 May 2021

[23] Nemabiome. 2021. https://www.nemabiome.ca/analysis.html. Accessed 20 May 2021

[24] White JR, Nagarajan N, Pop M (2009). Statistical methods for detecting differentially abundant features in clinical metagenomic samples. PLOS Comput Biol.

[25] Stromberg BE, Gasbarre LC, Waite A, Bechtol DT, Brown MS, Robinson NA (2012). *Cooperia punctata*: effect on cattle productivity?. Vet Parasitol.

[26] Aleuy OA, Kutz S (2020). Adaptations, life-history traits and ecological mechanisms of parasites to survive extremes and environmental unpredictability in the face of climate change. Int J Parasitol Parasites Wildl.

[27] Queiroz C, Levy M, Avramenko R, Redman E, Kearns K, Swain L (2020). The use of ITS-2 rDNA nemabiome metabarcoding to enhance anthelmintic resistance diagnosis and surveillance of ovine gastrointestinal nematodes. Int J Parasitol Drugs Drug Resist.

[28] Rose H, Caminade C, Bolajoko MB, Phelan P, van Dijk J, Baylis M (2016). Climate-driven changes to the spatio-temporal distribution of the parasitic nematode, *Haemonchus contortus*, in sheep in Europe. Glob Change Biol.

[29] Cordeiro MRC, Rotz A, Kroebel R, Beauchemin KA, Hunt D, Bittman S (2019). Prospects of forage production in northern regions under climate and land-use changes: a case-study of a dairy farm in Newfoundland, Canada. Agronomy.

[30] Gasbarre LC, Smith LL, Hoberg E, Pilitt PA (2009). Further characterization of a cattle nematode population with demonstrated resistance to current anthelmintics. Vet Parasitol.

[31] Gasbarre LC, Smith LL, Lichtenfels JR, Pilitt PA (2009). The identification of cattle nematode parasites resistant to multiple classes of anthelmintics in a commercial cattle population in the US. Vet Parasitol.

[32] De Seram E. Epidemiology and impact of gastrointestinal nematode infection in young beef cattle in western Canada [dissertation on the internet]. Saskatoon, Saskatchewan: University of Saskatchewan. 2021. https://harvest.usask.ca/handle/10388/13208.

